# Feasibility of an Interactive Coaching App to Enhance Post-concussion Outpatient Care

**DOI:** 10.3389/fmedt.2021.660540

**Published:** 2021-05-07

**Authors:** Dorothyann Curran, Julia Lauzon, Deanna Quon, Shawn Marshall

**Affiliations:** ^1^Clinical Epidemiology Program, Ottawa Hospital Research Institute (OHRI), Ottawa, ON, Canada; ^2^The Ottawa Hospital Rehabilitation Centre, Ottawa Hospital, Ottawa, ON, Canada; ^3^Faculty of Medicine, University of Ottawa, Ottawa, ON, Canada

**Keywords:** MTBI, post-concussion symptoms, rehabilitation, health coaching, smartphone app

## Abstract

**Objectives:** To determine the feasibility of patients to use a web-based health app for management of post-concussion (mTBI) symptoms in an out-patient setting.

**Participants:** Seven (7) patients who were referred to an outpatient specialist clinic (physiatry) with persisting symptoms following a concussion. Participants had to be 18 years of age or older and more than 3 months post injury.

**Design:** This was a prospective cohort study using a web-based platform for chronic disease management to guide patients in managing symptoms based on individual clinical recommendations. Each patient received weekly Symptom Management Plans created by a health coach and a physician specialist, designed to reinforce positive progress with clinical recommendations.

**Main Measures:** Adherence to tracking daily recommendations and symptoms (data collected through the web-interface), The Rivermead Post-Concussion Questionnaire (self report) and a Satisfaction Questionnaire (self report).

**Results:** Adherence to assigned clinical recommendations was close to 100%. Pre-post results on the patient reported outcome measure (Rivermead Post Concussion Tool) showed improvement for most patients in their experience of symptoms. The Satisfaction Questionnaire showed high rates of satisfaction with the App and the intervention in general.

**Conclusions:** Use of a web-based health app with a health coach is feasible in this patient population from both the patient and clinician perspective based on high adherence. There is also some evidence of improvement of symptoms with this intervention over time. Further exploration of the use of this type of intervention with post-concussion patients could potentially impact long-term outcomes.

## Introduction

Concussion, also known as mild traumatic brain injury (mTBI) accounts for between 70 and 90% of all adult TBI cases treated at hospital Emergency Rooms and/or admitted to hospitals worldwide (1, 2). In Ontario, the incidence rate is cited at 1.2% (3). While most people do recover from a concussion, many experience symptoms that persist beyond the usual 2–4 week timeframe that encompasses the acute post-injury recovery phase. In a cohort study involving concussion following motor collisions, 23% of individuals continued to have symptoms even 1-year post injury (2). The Guidelines for Concussion/mTBI and Persistent Symptoms: 3rd Ed (hereafter referred to as The Guidelines) defines persisting symptoms following concussion as; *A variety of physical, cognitive, emotional and behavioral symptoms that may endure for weeks or months following a concussion* (4). People with persisting symptoms usually exhibit multiple symptoms including cognitive fatigue, headaches, visual problems (including intolerance to screens), memory problems and sleep disorders, which can make it difficult to focus treatment. These persisting symptoms can become a potentially serious chronic health issue for previously active people, with various negative physical, social and economic impacts.

Specialized assessment can support symptom management by optimizing medication use and referring patients to appropriate allied health professionals or post-concussion specific programs. In the concussion clinic setting physicians spend significant time characterizing and prioritizing symptoms so that optimal treatments can be determined. However, there is good evidence to show that after patients leave the specialist appointment they exhibit poor adherence to recommended interventions and behavior changes and this can lead to continuing lack of improvement in symptoms (5–9).

Patient non-adherence to clinical recommendations for chronic conditions has been cited at anywhere from 25 to 90% (7, 9, 10). In many cases the main consequence of patient non-adherence is the lack of progress in treatment goals but in other circumstances health can be negatively impacted, especially with regard to medication use (5, 6, 11). In a recent article, Galey et al. (12) looked at patients with persisting vestibular symptoms post-concussion and divided them into three groups; high, moderate and low compliance to clinical recommendations. Those in the high compliance group were able to improve their clinical outcomes and reduce symptom severity in comparison to patients in the moderate and low groups (12). Encouraging adherence to appropriate health behavior is a critical component of improving health outcomes and patients often need support to modify their behavior and to recognize how their behavior influences their own health. Research has shown that strategies which seem to improve adherence, and therefore improve health gains for longer periods (5), typically involve individual or group counseling and include extended care sessions, skills training, and addressing barriers to implementing recommendations (8). Timely health care provider feedback and quality of physician communication also appear to be useful for influencing patient health behaviors (13–17). Based on these methods, if specialist physicians were able to bridge the gap between follow-up appointments by maintaining more frequent, high quality communication with patients over time, it could improve adherence to recommendations, enhance patient-centered care and ultimately improve outcomes for patients with persisting concussion symptoms.

There is increasing evidence that the use of internet-based interfaces can have positive impact on specific patient outcomes and patient satisfaction with treatments (10–18). Given that Canada has an extremely high rate of internet connectivity with almost 90% of the population able to access the internet in 2016 it is expected that most patients would find electronic interfaces for health care to be accessible (19). Online interaction can be done asynchronously by either party at any time, preventing the need for both the health care worker and patient to wait for a time that is convenient for both parties.

Olsen and Nesbitt (20), states that the health coaching relationship is “…patient-centered, with coaches providing education, feedback and support to enhance self-awareness, motivation, accountability and self-efficacy.” while the patients provide “…direction for learning and implementing changes” (20). Health coaching interventions using electronic tools have been shown to be feasible, to have positive impacts on health behaviors, and are viewed positively by numerous other ambulatory patient populations, such as people with chronic obstructive pulmonary disease (COPD), diabetes, coronary heart disease, inflammatory bowel disease (IBD) and obesity (11, 21–25). Applying the concepts of health coaching through an interactive app could manifest the success shown in other disease cohorts with post-concussion patients to more closely adhere to clinical recommendations and to experience better health outcomes. Recent articles have looked specifically at the use of apps to support people with traumatic brain injury, including persisting symptoms post-concussion (26–28), with the conclusion that further exploration of the potential of such apps is warranted.

There were two main objectives with this feasibility study, including (1) To determine whether we could successfully recruit patients with persisting symptoms post-concussion to use a web based application (App) and, (2) whether they would engage with the App and the coach within the study parameters we gave them for the time frame that we asked.

## Materials and Methods

This study was approved by the Ottawa Health Sciences Network Research Ethics Board prior to implementation. The web based app used for this study was NexJ Connected Wellness, created by NexJ Health Inc; https://www.nexjhealth.com/solutions/nexj-connected-wellness/ which has an interactive program interface allowing patients to record relevant health data, and communicate directly through a messaging feature with health care providers. The App, which is easily tailored to different patient population needs, allows both patients and health care providers to access the same data. Access to the App was provided by the Champlain Local Health Integration Network (LHIN) Home Care Innovation Center which provides health services and options for patient care in the community within a set geographic area.

Participants were recruited from the Post-Concussion Research Based Clinic at The Ottawa Hospital Rehabilitation Centre between June 14th and July 3rd, 2019. Inclusion criteria required patients to be confirmed to have had a prior concussion resulting in persisting physical and neuropsychological symptoms that required their referral to the specialist clinic from a community physician. Confirmation of a head trauma that resulted in <30 min loss of consciousness, <24 h post traumatic amnesia and/or alteration in mental state were recorded by a physiatrist or physician assistant through a comprehensive interview and physical exam in the specialist clinic. Other inclusion criteria for the study required people to be able to read and speak fluent English and sign an informed consent to participate. In total, 17 patients were approached for the study and 10 patients provided consent to participate. Of these 10 patients, three patients were unable to initiate the study; one experienced an unexpected life event, another was unable to be screened by the physician and another did not meet all inclusion criteria. There were seven patients who initiated the study and continued to completion (Table 1). Two physiatrists with significant experience in concussion/ brain injury rehabilitation were clinically responsible for care of the patients. A second-year medical student was employed to serve as the health coach.

**Table 1:** Patient demographics.

**Participant**	**Age**	**Sex**	**Mechanism of Injury**	**Time since injury (mo.)**	Target symptoms*
P1	37	M	Fall	6	Sleep Irritability Exercise tolerance
P2	45	M	MVA	13	Anxiety Sleep Exercise tolerance Screen tolerance
P3	31	F	MVA	8	Visual symptoms Anxiety
P4	62	F	MVA	12	Fatigue Sleep Anxiety
P5	50	F	Fall	15	Sleep Physical activity Headache
P6	39	F	Fall	6	Anxiety Headache Visual symptoms
P7	44	F	Fall	27	Headache Visual symptoms Sleep/Fatigue

*MVA, motor vehicle accident; mo., months*.

* *Patient trackers and target behaviors set out in the personalized Symptom Management Plans were created based on these target symptoms*.

Prior to participant recruitment, a professional ski coach with significant experience in coaching athletes using web-based technology and two previous concussion patients from the clinic served as consultants for the project. Each used the App for several days and provided feedback regarding which features they found most useful, the format of the questionnaires, and the App layout.

The clinic follows the recommendations of The Guidelines (4) which indicates the clinician should “…treat those symptoms that can be more easily managed and/or could delay recovery first…”. Therefore, the goals for each participant were set from their own consult summary where the physicians listed the main concerns that were discussed with the patient and options for treatment. Between 2 and 4 goals were identified for each participant.

**Figure 1:**
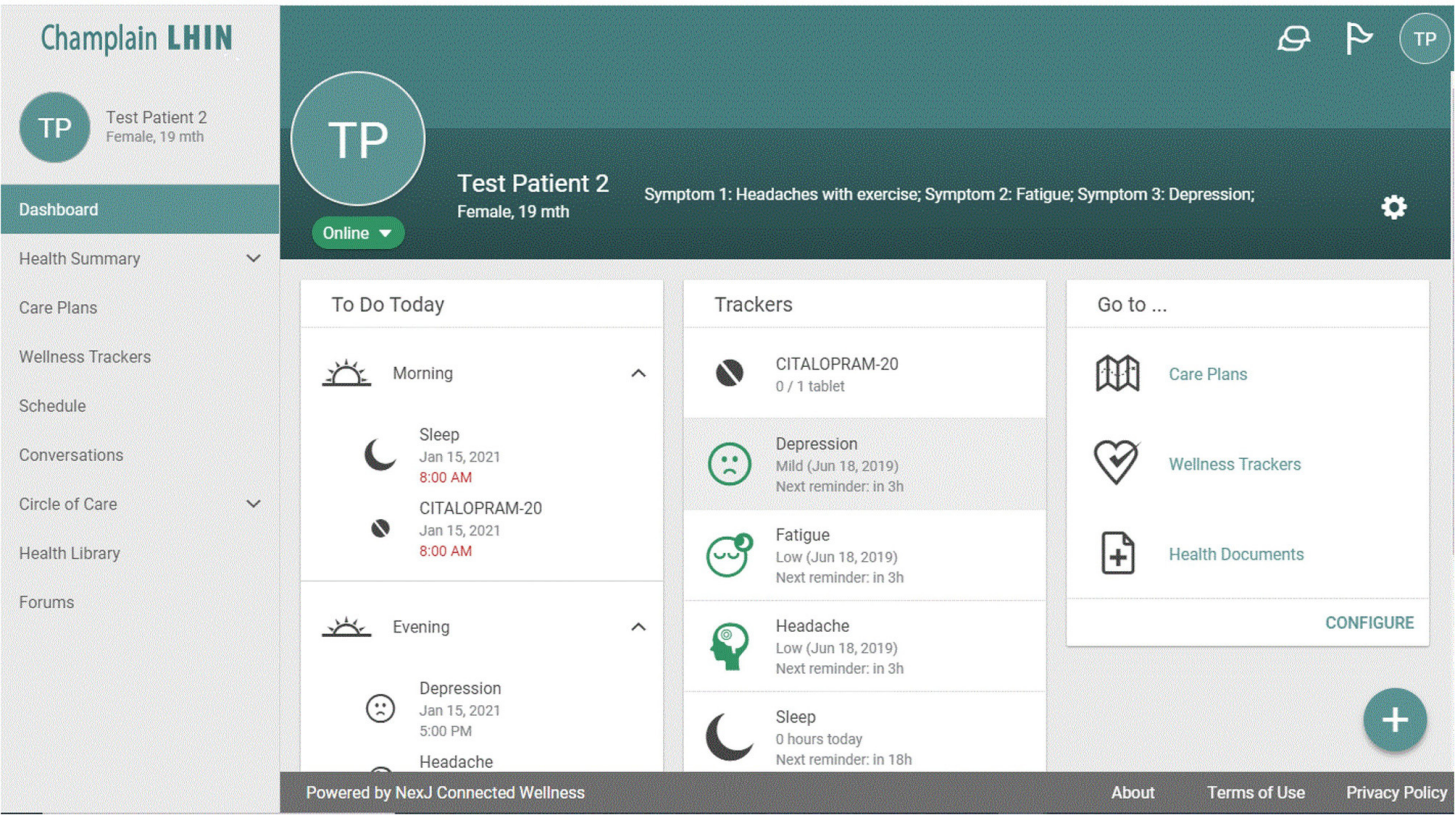
Profile page of the App interface. Basic daily reminders and instructions guide patients on this page.

Once they consented, participants were invited to join the App through the App itself by the health coach and a document was provided that detailed how to use the App for the purposes of the study. To ensure security, only a person who has administrative authority from a health care institution which has approved access to the App can invite people to use the App. The individual patient then creates their own password access to their profile. The health coach set up each participant's profile with their goals, and tracker information prior to sending the invitation. The health coach and physicians also had their own password protected access to the App with physicians having access only to their own patients. Once each participant logged on, the health coach was able to communicate with them directly through the App. For each participant, a personalized Symptom Management Plan, generated from the patient's goals and symptoms, was uploaded into the App by the health coach. The Symptom Management Plan provided details on what symptoms the participant was to track and what target behaviors they were to aim for Figure 1.

### Sample Personalized Symptom Management Plan

*Symptom 1: Track your sleep. Remember to practice good sleep hygiene. Track your use of Trazodone and whether you use 1/4 tablet or* ½ *tablet*.

*Symptom 2: Track your mood each day (irritability). Track your use of escitalopram (5 mg daily to start)*.

*Symptom 3: Aim to increase aerobic activity of your choice each day. Monitor your symptoms such that you push yourself but not to the point where you are affected for the rest of the day or the next day from doing your usual day-to-day activities*.

**Figure 2:**
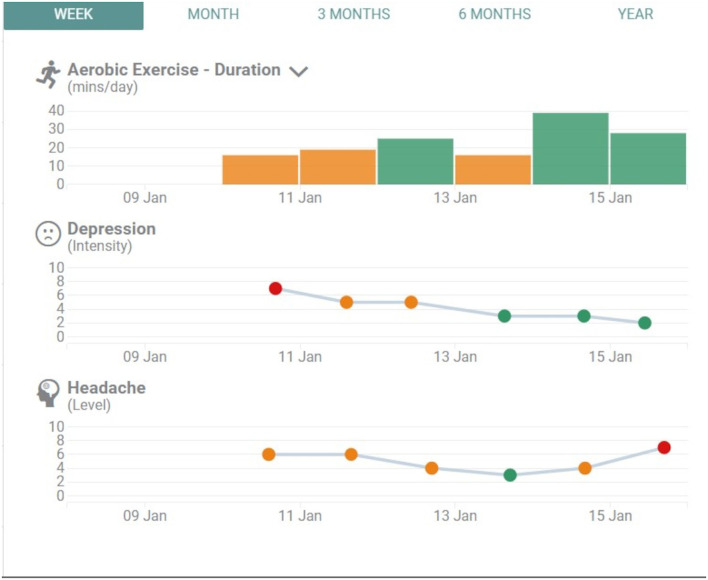
Example of 3 graphs comparing aerobic exercise, depression and headache over a 1-week period. Participants could generate an image showing any 3 symptoms/trackers over different blocks of time.

Participants were instructed to use the App each day to self-report their target symptoms (e.g., headache) and activities (e.g., assigned visual exercises). Each symptom tracker had a fan-shaped color wheel that ranged from green to red and a Likert scale from 0 to 10 that corresponded with each segment of the wheel. This offered both a colored visual (with green on one end indicating low intensity moving to red on the other end indicating high intensity) and a numeric scale [with low or good (0) moving to high or not good (10)], whereby the participant could gauge their symptom intensity or target achievement. They could also track their target activities digitally and rate intensity (e.g., try to do light exercise 20 min per day). Both types of “trackers” allowed the participant to record comments pertaining to their entries. Data from the trackers were also visible to the participants as a line graph or histogram over time (Figure 2). Participants could view the graph by week or by month to see progress over time and could also compare three graphs in the same view to observe different symptoms in relation to each other (Figure 2). Medication tracking was simply a yes/no response based on a prompt to take the medication at a scheduled time of day. All information in the App could be viewed by the participant, the health coach and the treating physician in real time. Participants were also provided patient resources that related to their symptoms (e.g., a document about sleep hygiene). These documents were uploaded into the App on an individual basis, and instructions to use the documents were included in their management plan. Most documents were sourced from the Ontario Neurotrauma Foundation website (29).

At the end of each week, a patient Progress Questionnaire was uploaded into the App by the health coach. The questionnaire was tailored to ask each participant about their specific goals and symptoms so that they could give their subjective opinion on their symptom control, how the Symptom Management Plan was working for them, and how well they were implementing their target behaviors. The health coach, in consultation with one of the treating physicians, generated a new Symptom Management Plan each week for every participant, based on participant feedback, the information in the daily trackers and any in-App communications over the course of the previous week. The new management plan was then posted on the same day each week for the participant to review and apply. This was done for each participant for 6 weeks.

The coach did not answer any medical questions in real time; all medical advice was communicated via the weekly Symptom Management Plans.

### Adherence

Although not all assigned trackers were required to be completed every day, tracking of symptom scores (e.g., sleep, anxiety) and medications was mandatory daily. The App would date and time stamp when people completed a tracking task, which allowed counts of completed tasks to be made for each patient. Tracker consistency of use was calculated by dividing the number of days that patients completed their required daily trackers, out of the expected 7 days. This value was calculated each week and converted to a percentage (Table 2). Higher percentage would indicate greater adherence to the clinical recommendations. The completion of the weekly questionnaire was scored either “yes” or “no” by recording if the participants completed the questionnaire by the requested date. The more frequently patients scored “yes,” the greater the adherence to study parameters (Table 2).

**Table 2:** Consistency of in-app tracking and progress questionnaire completion.

**Participant ID**	**Consistency of daily tracker use (%)**	**On-time progress questionnaire completion**
P1	97.6	5/5
P2	100.0	3/5
P3	76.8	5/5
P4	97.1	5/5
P5	98.4	4/5
P6	92.4	5/5
P7	98.6	5/5

*Tracker consistency of use was calculated by reviewing in-App time stamps and dividing the number of days that patients completed their required daily trackers, out of the expected 7 days. This value was calculated each week*.

*One patient had an extra week of App use so completed 6 progress questionnaires, however the data presented here represents the same 5 weeks for all participants*.

### Patient-Reported Outcome Measure (The Rivermead Post Concussion Questionnaire)

The Rivermead Post Concussion Questionnaire (30) is a patient reported outcome measure looking at functional outcome which is commonly used with this patient population. This tool was included to observe for any objective changes in study patients who were known to have prolonged symptoms post-concussion. It asks patients to rate 16 symptoms on a scale of 0–4 (none, mild, moderate, severe, and very severe) with four different categories of symptoms: vestibular, somatic/physical, cognitive, and affective. The maximum total score is 64. A total score of ≥16 is determined to be the optimal cut off point to distinguish between those with and without Post-Concussion Syndrome. Participants were given the questionnaire during their initial clinic appointment (Time 1) when they were recruited for the study, and at a follow up appointment (Time 2) which was 7-8 weeks later. A positive change, indicated by a lower symptom total score at Time 2, was sought by the team.

### End of Project Satisfaction Questionnaire

All participants completed a final project satisfaction questionnaire. The satisfaction questionnaire consisted of 16 questions with a five-point Likert scale and three open-ended questions. Higher scores indicated more positive evaluations. Sections for comments and general feedback were also provided.

### Messaging Data

The messaging data is a reflection of the extent of live interaction between the health coach and the participants. This information would give the study team an indication of how much interaction patients might undertake and also to understand what issues arose over the course of the project that would be addressed in this format.

### Statistics

Demographic data and Satisfaction Questionnaire data are described as counts and as mean (SD) while adherence data is described as percentage. Rivermead Questionnaire scores for two time points were examined using repeated measures *t*-Test (IBM SPSS Statistics 2016, Version 24).

## Results

### Patient Demographics

Seven participants (two males/five females) were followed for 5–6 weeks; one participant (P5) completed 6 weeks of tracking while the other participants completed 5 weeks. The average age was 44 and the average time since injury was 12 months.

This information was collected from patient charts once they agreed to participate in the study. Participants were not asked to provide information on their racial/ethnic background.

### Adherence

Adherence to using the requested trackers was very high with most participants close to 100% over the short time frame of the study. Almost all participants completed their weekly Progress Questionnaires on time each week, with only two participants completing them late. For those who completed questionnaires late, one participant submitted just one questionnaire late, while the other submitted two questionnaires late, with all late questionnaires being completed within 1-2 days of the original deadline, after reminders.

### Patient-Reported Outcome Measure (The Rivermead Post Concussion Questionnaire)

All participants completed the Rivermead Post Concussion Questionnaire in the clinic at Time 1. One participant did not complete a Time 2 questionnaire. Five participants reduced their scores (indicating improvement in function) by at least 10 points at Time 2 and one participant endorsed a one point increase in their score. Exploratory statistical comparison between the two time points did not show significance (*t* = 2.5, *p* = 0.055) however, the study was not specifically powered for this type of analysis.

### Participant Satisfaction

Weekly satisfaction scores for the Management Plans and perceived improvement of symptoms was collected for each participant, based on a 1-10 Likert scale for all 6 weeks of feedback. Over the course of the project, participants expressed higher satisfaction with the weekly management plan (M = 7.01, SD = 1.14) than with their improvement of symptoms (M = 5.8, SD = 1.75).

### End of Project Satisfaction Questionnaire

All participants completed the End of Project Satisfaction Questionnaire, which revealed overall satisfaction with both the App itself and the facilitation of the intervention by the health coach and their physician. There was mixed consensus from respondents regarding whether 4-6 weeks was enough time for this sort of intervention (Tables 3, 4).

**Table 3:** End of project patient satisfaction questionnaire responses.

	**Question**	**Mean (SD)**
1.	The App was easy to use.	4.6 (0.53)
2.	I was satisfied with the reliability of the App	4.6 (0.53)
3.	I was satisfied with the design of the App	4.1 (0.69)
4.	I was satisfied with the timeliness of advice that I received via the App.	4.9 (0.38)
5.	I was satisfied with the coaching that I received via the App.	4.7 (0.49)
6.	I was satisfied with the format of the feedback that I received in the Weekly Symptom Management Plans.	4.9 (0.38)
7.	Having a weekly management plan made it easier to follow my physician's instructions.	4.7 (0.49)
8.	It was easy to find the time to track my goals and symptoms daily, using the App	4.4 (0.79)
9.	During the time period of using the App, I felt as if I had more control over my health	4.1 (0.90)
10.	This App has increased my satisfaction with the management of my health	4.3 (0.76)
11.	This App helped me to improve my post-concussion symptoms.	4.0 (0.82)
12.	I believe that this type of App can be used by doctors to improve patient care.	4.9 (0.38)
13.	I believe that this type of App can be used by patients to improve their own health care.	4.7 (0.49)
14.	I believe that 4-6 weeks using the coaching App is enough to help people manage their symptoms	3.3 (1.38)
15.	I liked receiving new feedback every week in my Management Plan	4.9 (0.38)
16.	I think that receiving a Management Plan once every 2 weeks would be just as beneficial	4.3 (1.11)

*SD, standard deviation*.

*Satisfaction Questionnaire Likert scale; 1= strongly disagree; 2= disagree, 3= neither agree nor disagree, 4= agree, 5= strongly agree*.

**Table 4:** Qualitative findings (participant comments).

**What did you like best about the App?**
*P1: “Simplicity of use”.* *P2: “It was well-designed for the most part. And it really made me feel pressured to do the exercises, which is good!”* *P3: “The fact you can track your medication and important health information at one place.”* *P4: “Being able to map the symptoms visually. It helped show trends.”* *P5: “Follow up daily.”* *P6: “Once I learned how it worked, it was easy to navigate. I like tracking the symptoms that were more important to me.”* *P7: “Easy to track progress and symptoms—allowing the physician to see rather than just trying to explain it at an appointment.”*
**What could be improved about the App?**
*P1: “The App is good as is.”* *P2: “There were so many different tools/sections of the App that I didn't know where to look or how to use it. I'm more used to it now but still there are so many sections I didn't use or know about. 6 weeks isn't long to get used to such a complicated “App”… While the App seemed to be well-designed, I think it could be simplified.”* *P3: “Great app, but will need a little tweaking to make it simpler and easy to navigate”.* *P4: “I had other symptoms: headache, brain fog, difficulty in doing task, that I would have liked to see trends [for]”.* *P5: “One log for all symptoms”.* *P6: “Maybe make it a bit easier to learn from the beginning.”* *P7: “It can be a bit buggy at times. I found it a little cumbersome trying to remember exactly where everything was saved—Care Plans vs Health Documents, Health Questionnaires, etc. Those weren't intuitive and maybe would be better in one spot versus 2/3 spots.”*
**What did you like best about the communication received via the App?**
*P1: “Direct and clear.”* *P2: “It was good—straightforward. No comments for this.”* *P3: “I like the reminders.”* *P4: “Did not need to make an appointment. Could use it when convenient.”* *P5: “Personalized.”* *P6: “It wasn't much to read/understand/process.”* *P7: “Feedback was received quite quickly. There wasn't a need to wait for upcoming appointments”*.
**General participant comments:**
*P1: “The App was a useful tool. I've been keeping a daily journal that functioned similarly for a couple of months for tracking purposes, however, contact with health care professionals was useful and reassuring.”* *P2: “I was more successful than I would have been without the app/feedback…I was held accountable, which made me do the exercises regularly.”* *P3: N/A* *P4: “[The App] would be useful for self-management.”* *P5: “Thank you for your assistance and your feedback on my symptom management”.* *P6: “Personally, the hardest thing to realize is how much I have improved…Realizing that I didn't have a recurring headache for weeks as I entered that feedback was great. It helps to feel encouraged to keep on doing whatever works”*

### Messaging Data

In total, the health coach sent 167 messages to participants, and received 89 messages from participants, over the 6 weeks. The participants sent an average of 12.7 messages total over the 6 week period (~2/week, range of 4-25 messages) and each participant received an average of 23.8 messages (~4/week, range of 15-40 messages). The main purpose of the messaging was for reminders, notifications or administrative clarifications whereas communication for treatment purposes was directed through the weekly Symptom Management Plan (Tables 3, 4).

## Discussion

As a component of this feasibility study our first objective was to determine if participants could be recruited. To our knowledge, there is no pre-determined rate of recruitment that is accepted as appropriate for specific study designs and recruitment rates vary widely in research. For our purposes, the success rate of recruiting ~1/3 of the people approached will serve to inform methodology for a larger trial design. This recruitment rate would be acceptable for our clinic to manage a randomized control trial based on the existing clinic processes. One of our concerns was also the potential barrier that using devices could present for our patients since it was anticipated that many patients may not be tolerant of the screen time demands. Many patients with persisting symptoms post-concussion experience difficulty reading text on screens as part of their visual issues following injury so using an App could have been a deterrent to participation. Although improving tolerance for screen time/reading was noted in the charts of three participants as a goal to be addressed, almost all the patients managed to use the App as suggested. Despite efforts to keep the navigation simple, feedback from a couple of participants included comments relating to difficulty finding their way around the App. Despite these comments, the participants rated the App's simplicity of use quite high revealing that these technical inadequacies did not grossly affect their ability to use the App.

With regard to the second goal of the study, in order to maintain a set schedule for the physician and health coach to review responses, participants needed to complete their daily tracking and the Weekly Progress Questionnaires by a certain date each week. If the participants were unable to complete the questionnaire on time, the weekly recommendations sent by the health coach would be based on incomplete data. The fact that the participants were willing to engage at such a high level of adherence allowed the physician and health coach to review complete data each week and make individualized recommendations for each individual.

Weekly monitoring of patients allowed the physician and health coach to suggest modifications of behaviors, reinforce certain recommendations, and to make medication adjustments, in a timely fashion. Some goals and behaviors originally set by the patients and physician were modified, and some were added. For 2 participants, medications were prescribed to address lack of progress with symptoms, something which would only have been discovered otherwise at the next face-to-face appointment (anywhere between 1 and 4 months for these patients). Regular monitoring of the participants' progress with the clinical recommendations meant that both parties had a better understanding of the treatment impact, and the typical delay imposed by in-person appointments was mitigated. This allowed patients to make progress at their own pace and increased their awareness and responsibility for their own health, as was reflected in their comments.

Based on the results of this project, the application of this type of intervention with this patient population is very encouraging. Our participants also reflected some key features on the impact of health coaching-type interventions as various other chronic patient populations. For example, literature reviews on maintenance of behavior change by Olsen and Nesbitt (20) and Fjeldsoe et al. (31) found that interventions lasting 6 months or longer and with higher numbers of intervention contacts resulted in greater likelihood of achieving maintenance of behavior change. In our study, 3/7 patients felt that the App should be used for a longer time frame than the 6 weeks that they committed to. In fact, 2 people continued to use the App independently even after the study ended.

Having the physician directly involved in monitoring the progress of patients over the 6 weeks of the study proved key to ensuring that participants were able to make progress on their goals. This increased level of communication between patients and providers over an extended time period optimizes health information provision to patients (32) and could potentially result in long-term positive health outcomes for patients (33). In this context the timeliness of advice, the format of feedback in the Symptom Management Plan and receiving new weekly feedback were the most highly endorsed questions in the End of Project Satisfaction Questionnaire. All participants agreed that having individualized resources (websites, information sheets tailored to their symptoms) available in the App was beneficial in helping them manage their symptoms.

Our participants produced similar comments as participants in other studies regarding the positive impact that the App had on their accountability and responsibility to complete their treatment recommendations (22, 23, 34).

One advantage of the App is the ability to have patients monitor their own progress and reflect on how their behavior changes have impacted their symptoms over time. One of the unique features of the App were the graphs, which allowed participants to track their progress day by day. The visual presentation, and the ability to manipulate it to view more than one symptom seemed to be very engaging for participants.

Using the graphs as references for both positive and negative trends in patient progress, made feedback more concrete. The graphs also showed patterns over time that would not be as easily discernable with another symptom tracking mechanism such as a daily diary.

When Nieuwlaat et al. (35) completed a Cochrane literature review of Randomized Control Trials (RCTs) looking at techniques to improve patient adherence to self-administered medications the use of electronic tools as study interventions was almost non-existent in their review. This suggests that prior to 2013 electronic support options for clinical adherence were very limited. The impact on patient care and outcomes in relation to the burgeoning expansion of features available for this type of technologic intervention should continue to be evaluated. Features of the newer app technologies such as streamlined visual presentations, real-time recording of data and greater personalization have made health apps even more appealing to the population. The incorporation of this type of intervention into outpatient clinics could mitigate issues from lack of clinician access or redistribution of health care resources while maintaining consistency in treatment toward improved health outcomes for many patients.

There are some limitations with this project. The App was originally designed to be a more directive tool, not designed for health coaching of a patient population having such diverse symptoms and requirements for tracking many different health behaviors. Although the project team adapted the App relatively well for the study's purposes, by limiting the number of health goals/targets and having the health coach work through the set-up with participants, there were limitations that were evident in the participant's feedback. This project also utilized only specific aspects of the App and there were features that the team chose not to use in order to limit the demands on participants and physicians for the short duration of the study. For example, participants were not instructed to invite family members or their general practitioner to join the App, which is a feature that the App allows to provide further social and clinical support to the patient.

For the execution of this feasibility study, the coach was a medical student who had not met any of the participants in person. Having the coach be a part of the patient's healthcare team (physician assistant, occupational therapist, etc.) could have an impact on outcomes and would facilitate smoother processes in the clinic itself.

Further research could be done to clarify whether there is a clinically relevant improvement in symptoms on the Rivermead for patients who undergo this intervention (36). Functional change could also be assessed using a tool such as the Concussion Recovery Questionnaire (37). Clinically, it was not a part of this study to ensure that patients reached specific goals but to facilitate them working on goals after they met with the specialist. Future work could examine more closely the relationship between use of the App and achievement of patient goals, especially over a longer time frame.

The results of this study demonstrate the feasibility for the use of a health App and online health coaching for patients with persisting symptoms post-concussion in an outpatient clinic. This intervention allows patients to reflect on their health and the impact of treatments while also providing timely feedback from health care providers. Further study of both this tool and method of patient monitoring is needed.

## Data Availability Statement

The raw data supporting the conclusions of this article will be made available by the authors, without undue reservation.

## Ethics Statement

This study was approved by the Ottawa Health Sciences Network Research Ethics Board prior to implementation. The patients/participants provided their written informed consent to participate in this study.

## Author Contributions

DC and JL contributed to the design of the study, conducted analyses, wrote and edited the article. DQ contributed to the design of the study and reviewed edits to the article. SM conceived and designed the study and edited the article. All authors contributed to the article and approved the submitted version.

## Conflict of Interest

The authors declare that the research was conducted in the absence of any commercial or financial relationships that could be construed as a potential conflict of interest.
